# PB.26: Significance of flat epithelial atypia at image guided breast biopsy

**DOI:** 10.1186/bcr3526

**Published:** 2013-11-08

**Authors:** S Dani, S Sudderuddin, G Ralleigh, N Zaman, A Gupta, N Barrett, D Cunningham, B Faissola, S Comitis, W Svensson, A Lim, R Williamson, V Stewart

**Affiliations:** 1 Imperial College Healthcare NHS Trust, London, UK

## Introduction

The term flat epithelial atypia (FEA) was introduced in 2003 by the WHO working group on the Pathology & Genetics committee of tumours of the breast. Previously known as columnar cell change with atypia, these lesions usually present as microcalcification and although there is some evidence to suggest that FEA represents a precursor lesion in the spectrum of low-grade breast neoplasia, there are limited data concerning the ultimate clinical impact and optimum management strategy. The aim of this study was to determine the radiological characteristics of FEA and final histology following surgical excision at our centre.

## Methods

Retrospective review of 950 consecutive image-guided core and vacuum biopsies performed over an 8-year period revealed 162 lesions with FEA as the most prominent pathologic entity; concomitant lobular neoplasia, ADH or malignancies were excluded. The radiologic characteristics of FEA/CCC lesions were assessed with respect to size, morphology and multifocality at presentation. Biopsy technique and final histology of these lesions following surgical excision were recorded.

## Results

The rate of upgrade to a more significant pathology was 10% (on provisional data, complete results will be presented) with significant pathology being DCIS of varying grade. Microcalcification was the most common mammographic sign.

## Conclusion

Surgical excision of FEA at our centre has resulted in a low rate of upgrade to malignancy supporting current thinking that some lesions may be managed by vacuum excision, thus reducing the surgical diagnostic excision rate.

